# Summarized Report of the Conference on Surgery in Battle Areas

**Published:** 1918-10

**Authors:** G. W. Crile


VOL. II, No. 3 PARIS OCTOBER 1918

### WAR MEDICINE

Published by the American Red Cross Society in France
for the
Medical Officers of the American Expeditionary Forces

### RESEARCH SOCIETY REPORTS

### THE TENTH SESSION OF THE RESEARCH SOCIETY OF THE AMERICAN RED CROSS SOCIETY IN FRANCE

September 6 and 7, 1918, at the Hotel Continental, Paris.

*The first meeting of the Session, September 6 at 2 : 00 P.M., was devoted to a Conference on Surgery in Battle Areas, led by Lieutenant-Colonel Crile. It was followed by a demonstration of splint adjustment by Lieutenant-Colonel Joel Goldthwait, M. R. C.*

*The second meeting, September 7 at 9 : 30 A.M., was devoted to a discussion of Acute Respiratory Infections. Papers were read by Colonel W. W. O. Beveridge, A. D. M. S. Sanitation, B. E. F., Médecin-Major Borel of the Service de Santé, Major Haven Emerson, M. R. C., and Major H. Zinsser, M. R. C.*

*The third meeting, September 7 at 2 : 00 P.M., was devoted to a discussion of Field Sanitation. Papers were read by Médecin-Major Octave Monod, Major F. P. Josceline, A. D. M. S. Sanitation, B. E. F., Colonel Henry A. Shaw, M. C., and Colonel Gressinger, M. C..*

### SUMMARIZED REPORT OF THE CONFERENCE ON SURGERY IN BATTLE AREAS

Leader, Lieutenant-Colonel G. W. CRILE, M.R.C.

Friday, September 6, 1918.

#### INTRODUCTION.

The great value of the Conference was owing to the fullest participation with American officers of certain military surgeons of the widest experience — including the clinical chiefs for both France and England. These distinguished officers gave to our cause their counsel and advice as freely and as enthusiastically as if it were for their own armies. Among these were Professor Tuffier, General Sir Anthony Bowlby, Colonel Gask, Colonel Richards, Colonel Soltau, Major Lockwood, Captain Betz and Captain Dehelly.

The conclusions of this Conference were slightly modified in a later Conference with a Committee appointed by the Chief Consultant, A. E. F. The following is the final conclusion.

### I. Problems Relating to Organization.

1. **What should be the personnel of a standard surgical team?**

A. — (*a*) Surgeon.

(*b*) Assistant surgeon.

(*c*) Nurse assistant.

(*d*) Anesthetist (Nurse or M. O.).

(*e*) Two theater orderlies.

(*f*) One striker.

*Comment :*

(*a*) and (*b*) *Surgeon and Assistant surgeon* : Every surgeon should have an assistant surgeon for two reasons : first, to train the younger men to become competent surgeons; and second, to facilitate work. The assistant surgeon of today is the surgeon of tomorrow. The relation between the surgeon and his assistant should be that of pupil and teacher. A nurse or an orderly may be trained to be as useful a technical assistant as an officer.

When there is a sufficient number of trained surgeons, the nurse assistants or orderly assistants may be used to replace the medical assistants.

(c) *Nurse Assistant:* Were it not for the necessity of training new surgeons, the nurse assistant answers every purpose.

(d) *Anesthetist* : Preferably a nurse, for

1. Each nurse anesthetist adds an officer to the medical corps.
2. The nurse anesthetist has proven satisfactory to the surgeon.
3. The nurse anesthetists should be trained just as surgeons are trained — by acting as pupils to skilled anesthetists. Skilled anesthetists should have pupils until there are a sufficient number of trained anesthetists for the army. Women physicians, trained in administering anesthetics, may be employed. The *degree* of M. D. does not confer qualification to give anesthetics. Every anesthetist, medical or non-medical, should receive adequate training at the expense of the teacher, not at the expense of the patient.

(e) *Orderlies:* Each team should have two theater orderlies, and, if there is shortage of personnel in the forward area, four nursing orderlies and two bearers may be added to a team to reinforce the personnel of the forward unit. Surgeons are of little use if patients cannot be carried. An organization may break at the point of enlisted personnel, as well as at the point of surgical personnel. The quality of work and the quantity of work are dependent on a well-balanced personnel.

(f) *Striker:* The striker is an accessory, but the surgeon's time can be better occupied than in taking care of his own quarters. Disorderly quarters, mess-room and premises adversely affect the work of an organization.

2. **How many tables shall be allotted to each team?**

A. — In ordinary cases, two; in lightly wounded, three or even more. In rush periods, patients need not be removed from the stretcher; they may be operated on the stretcher resting on the table. But few metal tables are required — anything that will support a stretcher will answer. In periods of rush, the time and labor of orderlies must be conserved as well as the time and labor of the surgeon.

3. **What is the best arrangement of hours for a considerable pull?**

A. — When the fight opens and the wounded begin to come in, all surgeons go on duty for the first 4 to 6 hours; then they begin to go off in relays of eight-hour and twelve-hour operating periods.

The following plan has worked out satisfactorily in a longer pull: four hours on and four hours off, excepting the night team, which works straight from midnight to 8:00 a.m. By having two breakfasts, two lunches and two dinners, an hour apart, and at the same hours for surgeons, nurses, and orderlies of the same team, the operating tables may then run continuously.

| *First Team.* | | *Second Team.* | | *Third Team.* | |
|---|---|---|---|---|---|
| On. | Off. | On. | Off. | On. | Off. |
| 8 a.m. - 12 N. | 12 N. - 4 p m. | 12 N. - 4 p.m. | 4 p.m. - 8 p.m. | 12 M. - 8 a.m. | 8 a.m. - 12 N. |
| 4 p.m. - 8 p.m. | 8 p.m. - 8 a.m. | 8 p.m. - 12 M. | 12 M. - 12 N. | | |

4. **What is the best means of transport of teams?**

A. — Ambulance.

5. **Shall teams furnish any instrument or apparatus?**

A. Teams should be self-contained as regards the commonly used instruments, unless the hospital to be reinforced has an adequate excess of instruments.

6. **What shall be the proportion of X-ray operators?**

A. — For each three teams; an operator for the day and an operator for the night would seem to provide sufficient X-ray service.

7. **What shall be the proportion of nurses, not attached to teams, in the operating room?**

A. — Two nurses to supervise the theater in the day and two in the night, for each six teams.

8. **Shall each surgeon be responsible for the after-care of his cases?**

A. — Yes, as far as possible.

9. **Shall there be a day and night chief of surgical service?**

A. — Yes, the chief of surgical service by day, and the second surgeon by night.

10. **Under whose direction shall the resuscitation team work?**

A. — The resuscitation team will work under the general direction of, and in close co-operation with, the operating surgeons. It shall consist of two medical men, two nurses, and two orderlies.

11. **Shall abundant reserves of teams be in readiness?**

A. — There must be kept as many teams in reserve as possible — teams trained to work together. The teams should be promptly exchanged between the front area and the base according to need.

12. **What is the most available type of mobile unit for emergency reinforcements?**

A. — As far as possible mobile hospitals — mobile operating units. When advancing, have reserve mobile units ready, and leap-frog these past the units in operation. Operate, evacuate and leap-frog again. If these mobile units are not available in sufficient number, then make use of the field hospital.

As a policy, except in emergencies, the field hospital should not function as an operating center.

The mobile unit must be *flexible*, must remain *mobile*, must be *expansible* in its operating capacity. Its primary job is to afford great facility for operating. Evacuate every operated case as early as possible. As a rule early evacuation after operation is better than in the third or fourth day, which are the critical days for infection. It is the great virtue of gas and oxygen that evacuations are hastened.

13. **In periods of great activity what cases can be evacuated unoperated from the evacuation hospitals?**

A. — During periods of great activity, when it is impossible to make complete operations at the evacuation hospitals within a safe period of time, the following types should be promptly sent by pre-operation train to the nearest base :

(1) Perforating wounds with punctiform orifices.

(2) Superficial wounds, and those deeper wounds not extensively involving the musculature of the calf, thigh, buttock, and shoulder areas.

(3) Wounds of the face, hands, and feet, not involving tarsus and deep plantar fascia.

(4) Fractures of bones caused by rifle and machine-gun bullets with through and through wounds, and without extensive comminution, marked displacements, or marked swelling.

### II. Problems Relating to War Wounds.

1. **In cases suitable for primary closure, it has heretofore been agreed that patients should remain under the care of the operating surgeon until there is sound healing. Are there any new facts suggesting a modification of this principle?**

A. — Wounds should not be closed unless they can be continuously under the care of the operating surgeon until the wound is healed. There are these exceptions: heads, joints, sucking wounds of the chest, abdomens (in chest and joints it may be well to leave skin unsutured). These should be closed, and if they cannot be held until healed, they should be sent on at once rather than evacuated in the third or fourth day when infection is apt to be spreading. If a sutured wound is to be moved at all, it should be moved promptly.

2. **If a suturable case must be evacuated, and if there is opportunity for making débridement before evacuation, shall the surgeon introduce stitches, leaving them untied, or shall the wound be left wide open without stitches placed?**

A. — Put in no stitches unless they are to be tied at once. Untied stitches in a well debrided wound may become infected; infected stitches delay subsequent closure. Inserted, untied stitches are a handicap, not a help, for a delayed primary wound should have its first dressing under anesthesia in the theatre — preferably gas and oxygen anesthesia — and the entire field cleansed under anesthesia, as if for performing an aseptic operation; then and only then should the gauze dressing be removed and the wound immediately closed. Redressing such wounds in the wards usually leads to infection.

3. **Shall debrided wounds be protected by sterile gauze during evacuation? Or shall an aseptic dressing be applied? If so, what antiseptic?**

A. — Dry sterile gauze; paraffined gauze; and, if there is a probability of two or more days' delay, the peculiar property of flavine which inhibits bacterial growth is an advantage; hence, in such cases, gauze wet with 1/5000 flavine is good. Studies are being undertaken with other methods which may modify the foregoing.

4. **Shall wounds be packed with gauze?**

A. — Loosely dressed with gauze, yes; *packed* with gauze, never. Dry gauze should be placed in contact with every part of the raw surface, for it absorbs toxins, bacteria and wound secretions, hence aids the wound in its defense against infection.

5. **Shall splints be placed on limbs having wounds of the soft parts only?**

A. — In extensive wounds, yes, not only because they provide comfort and physiologic rest, but also, if nerves or tendons have been severed, they are less displaced.

6. **Is a bacteriologic examination of the wound required in making primary suture during the period of contamination, which usually lasts from 10 to 12 hours?**

A. — No. Clinical judgment is sufficient in primary suture.

7. **If, owing to rush, no surgical treatment of a wound can be given before evacuation, shall the wound be covered with dry sterile gauze only? Shall any antiseptic be used? If the latter, what antiseptic?**

A. — Make no instrumental, no digital examination of the wound. Place no drain tubes, no gauze, no foreign body in the wound. Apply dry sterile gauze dressing. It may be worth while to apply alcohol, picric acid, or iodin on the skin only.

8. **If there is a larger number of wounded than the surgeon can give a complete débridement, shall he complete as many as he can, leaving the remainder of the wounded unoperated, or should he perform an incomplete operation on every wounded man?**

A. — The adage that "Anything worth doing at all is worth doing well" applies here. Under these circumstances, the surgeon should give his time to those patients on whom operation is likely to result in recovery; and those who, if unoperated, are not safe for evacuation. In stress, the surgeon should do only work with greatest net profit.

It has been shown that wounds of the soft parts, including compound fractures, may be remarkably well reclaimed up to and into the third day. The reclamation is made through complete revision, just as in the wounds at the front — just as acute appendicitis is reclaimed up to the period of new tissue formation and abscess.

Do well as much as possible; evacuate the rest.

At the beginning of a fight, there is a tendency to hold too many cases at the front hospitals. Evacuate as quickly as possible. Evacuate the earliest cases liberally. "Don't be selfish and try to do them all in the front area. Remember the surgeon back at the base is ready for work. The cases must be sent back in any event. Send all that can travel. Keep abdomens, chests, joints, amputations, and shock cases."

An added reason for a liberal evacuation of unoperated cases at the opening of the battle is that a larger number of surgeons will thereby enable the greatest number of patients to receive prompt operation.

9. **In battle stress, shall the surgeon with ripest experience and most mature judgment do operations, or shall he utilize his experience and judgment in making important decisions; in advising less experienced operators; in directing treatment of critical cases; in deciding the schedule of operating — in short, acting as a surgical manager, operating with his head, not his hands?**

A. — There should be a surgical manager, not necessarily the best technical surgeon, but the man with the best judgment.

10. **Shall the wounded receive morphia?**

A. — Give morphia for pain and for great restlessness. If time permits, give quarter grains and repeat rather than start with half grain. Always ticket the time and the dose on the man.

### III. Problems Relating to the Lightly Wounded.

1. **Shall the lightly wounded be segregated and dealt with independently of the seriously wounded?**

A. — In large fights, the lightly wounded should be separated at the triage and sent directly to an advanced hospital especially set aside for their treatment, or sent to the base at once.

2. **Shall they claim the attention of the best surgeons under the best conditions, or otherwise?**

*A.* — The organization for the care of the lightly wounded should be made by one of the best surgeons, although the actual work may be done by those of less experience.

